# Clinical, hematologic, and inflammatory factors associated with psoriatic arthritis in a Chinese hospital-based study

**DOI:** 10.3389/fimmu.2026.1928389

**Published:** 2026-09-15

**Authors:** Yingying Sun, Shiqi Fu, Jianjun Yan, Ningning Dang

**Affiliations:** Department of Dermatology, Shandong Provincial Hospital Affiliated to Shandong First Medical University, Jinan, Shandong, China

**Keywords:** associated factors, musculoskeletal ultrasound, psoriasis, psoriatic arthritis, subclinical psoriatic arthritis

## Abstract

**Background:**

Psoriatic arthritis (PsA) develops in up to approximately 30% of patients with psoriasis (PsO) and can cause irreversible joint damage. For dermatologists, identifying factors independently associated with PsA may help identify patients who may benefit from earlier musculoskeletal monitoring and specialist referral.

**Objective:**

To identify demographic, clinical, and laboratory factors independently associated with clinical and subclinical PsA in patients with psoriasis.

**Methods:**

This retrospective study at Shandong Provincial Hospital included 75 clinical PsA, 22 subclinical PsA, and 95 PsO patients. Subclinical PsA was applied as an operational label only: 21 of these 22 patients met the symptom-based criterion alone and 1 met both the symptom-based and imaging-based criteria. Separate multivariable logistic regression models were fitted for clinical PsA versus PsO and subclinical PsA versus PsO, checked for multicollinearity, with C-reactive protein (CRP) log-transformed and platelet (PLT) count expressed per 50×10^9^/L. Age and sex were force-entered but were not retained after backward elimination, so each estimate is adjusted for the other variables retained in the same model. Model performance was assessed by the Hosmer-Lemeshow test, area under the receiver operating characteristic curve (AUC), and Nagelkerke R².

**Results:**

In clinical PsA, lower hemoglobin (adjusted OR 0.75 per 10 g/L increment), higher PLT count (adjusted OR 1.39 per 50×10^9^/L increment), and higher CRP (adjusted OR 1.78 per doubling) were independently associated with PsA (all *p* < 0.01; AUC 0.859). In subclinical PsA, hemoglobin and CRP showed comparable independent associations (AUC 0.878); PLT count could not be reliably assessed given the small subgroup (n = 22). Clinical PsA showed more frequent tenosynovitis, synovitis, synovial thickening, and joint effusion on ultrasound than subclinical PsA (*p* < 0.05).

**Conclusion:**

Lower hemoglobin and elevated CRP were consistently associated with PsA across both subgroups. Routine hematologic and inflammatory markers, combined with musculoskeletal ultrasound (MSUS), may help dermatologists identify psoriasis patients warranting earlier rheumatological referral; prospective studies are needed to confirm predictive value.

## Background

Psoriatic arthritis (PsA) is a chronic and inflammatory disease, characterized by arthritis or enthesitis of peripheral and axial skeleton (1). Approximately 20% to 30% of patients with PsO develop PsA, a condition characterized by significant clinical and course heterogeneity. Notably, a considerable proportion of patients progress to erosive and debilitating forms of arthritis, resulting in substantial morbidity and functional disability (2, 3). These musculoskeletal inflammatory manifestations share common pathophysiological links to skin disease in PsO, but the severity of skin lesions and arthritis in patients are not parallel, with skin lesions manifesting before arthritis symptoms in about 75% of PsA patients (4, 5). Over time, these inflammatory processes may culminate in irreversible joint damage and functional disability (6), thereby underscoring the critical importance of early identification and timely targeted intervention. Recent evidence highlights a subclinical PsA phase characterized by occult joint inflammation or morphological changes detectable via musculoskeletal ultrasound (MSUS); initiating intervention during this critical window is paramount to preventing irreversible damage and optimizing long-term quality of life (7). In this study, we conducted a multidimensional evaluation of environmental exposures, comorbidity profiles, and laboratory markers unique to the Chinese population. This study characterizes factors associated with musculoskeletal involvement at the time of assessment and thereby support the early identification and timely therapeutic intervention of PsA.

## Methods

### Study design

This retrospective study includes clinical PsA, subclinical PsA and PsO groups. All patients were selected from Shandong Provincial Hospital Affiliated to Shandong First Medical University, from January 2018 to October 2025 and fulfilled the inclusion criteria outlined below.

### Inclusion and exclusion criteria

Patients with PsO who were hospitalized at Shandong Provincial Hospital Affiliated to Shandong First Medical University between January 1, 2018, and October 31, 2025. The diagnosis of PsO was confirmed by dermatologists based on clinical characteristics of skin lesions or histopathological examination results. Exclusion Criteria: (1) Patients with pustular PsO or erythrodermic PsO; (2) Degenerative joint diseases; (3) Other inflammatory arthritis, such as rheumatoid arthritis; (4) Lack of essential clinical records required for this research.

### Grouping method

Clinical and subclinical musculoskeletal phenotypes were operationally defined with reference to recent EULAR points to consider on the psoriatic disease spectrum (8): (1) PsO group: patients with PsO who had no musculoskeletal symptoms, including joint pain, morning stiffness, or joint swelling, and showed no clinical or radiographic evidence of arthritis. (2) Subclinical PsA group: refers to patients with PsO who exhibit early musculoskeletal manifestations that do not yet fulfill the formal diagnostic criteria for clinically overt PsA. In this study, subclinical PsA functions solely as an operational label to describe psoriasis patients with musculoskeletal symptoms or ultrasound markers who do not meet PsA classification criteria. It is not a standardized diagnostic code, nor does it denote a proven pre-arthritic stage. Patients were classified into this group if they met either of the following criteria: (i) nonspecific musculoskeletal symptoms, particularly occult arthralgia, which may be accompanied by morning stiffness or functional limitation, in the absence of clinically apparent joint swelling or tenderness; or (ii) MSUS evidence of synovio-entheseal inflammation, defined as the presence of any of the following: synovitis, tenosynovitis, synovial thickening, or joint effusion, also in the absence of clinically apparent arthritis. Patients did not need to fulfill both criteria simultaneously; either criterion alone was sufficient for classification as subclinical PsA. In the present cohort, 21 of the 22 patients (95.5%) fulfilled the symptom-based criterion only, 1 patient (4.5%) fulfilled both the symptom-based and the imaging-based criteria (musculoskeletal symptoms together with synovial thickening on MSUS), and no patient fulfilled the imaging-based criterion alone. This inclusive approach was adopted because no consensus operational definition currently exists across the literature — some cohorts ascertain subclinical/at-risk PsA using musculoskeletal symptoms alone, others using imaging abnormalities alone — and was intended to maximize sensitivity for detecting early joint involvement, consistent with the study’s aim of characterizing systemic inflammatory correlates across the full disease spectrum rather than validating a diagnostic instrument. We acknowledge this either/or criterion is broader than approaches requiring concurrent symptomatic and imaging abnormalities, and may therefore capture a more heterogeneous population. (3) Clinical PsA group: refers to the presence of clinically evident synovitis in patients with PsO, manifested by palpable joint swelling and/or digit swelling attributable to dactylitis, which cannot be explained by alternative diagnoses, including osteoarthritis, gout, or infectious arthritis. All patients assigned to the clinical PsA group were subsequently reviewed against the Classification Criteria for Psoriatic Arthritis (CASPAR), and all patients fulfilled the CASPAR criteria (score ≥ 3). Group assignment was made by 2 dermatologists with experience in psoriatic disease, in consultation with a rheumatologist when the diagnosis was uncertain; disagreements were resolved by consensus.

### Data acquisition and clinical information

Data on patient demographics, clinical characteristics, joint involvement, imaging examination results, and laboratory examinations were collected from the hospital information system. The missing or unverified information was uniformly obtained through telephone follow-up conducted by an experienced dermatologist, primarily to confirm historical information such as the onset timing of musculoskeletal symptoms and family history of psoriasis.

### Specimen collection and laboratory examination

Peripheral venous blood was also collected from all patients within 24 hours of admission, and further blood samples were collected as needed. The retrospective data collection encompassed a comprehensive panel of laboratory parameters: hematological indices (white blood cell count (WBC), hemoglobin (HB), platelet count (PLT), and differential leukocyte percentages including lymphocyte percentage (LYMPH%), monocyte percentage (MONO%), and neutrophil percentage (NEUT%); systemic inflammatory markers (C-reactive protein (CRP) and erythrocyte sedimentation rate (ESR)); liver function indicators (alanine aminotransferase (ALT), aspartate aminotransferase (AST), gamma-glutamyl transferase (GGT), and albumin (ALB)); and metabolic and renal function markers glucose (GLU), uric acid (URIC), and creatinine (CREA). For the same laboratory indicator in the same patient, the result closest to the time of admission was used.

### Musculoskeletal ultrasound (MSUS) examinations

MSUS was performed using an Aplio i800 ultrasound system (Canon Medical Systems) equipped with an i24LX8 linear array transducer. All examinations were performed by a single sonographer with more than 5 years of clinical experience in musculoskeletal ultrasonography. MSUS was performed on peripheral joints with clinical symptoms or a history of involvement, including the interphalangeal joints, metacarpophalangeal joints, wrist, elbow, shoulder, knee, ankle, toe interphalangeal joints, and metatarsophalangeal joints. Both grey-scale and power Doppler modes were used to assess the synovium, entheses, and tendon sheaths, with reference to the consensus-based definitions of the EULAR-OMERACT Ultrasound Task Force (9). The following MSUS findings were recorded: joint effusion, synovial thickening, increased intra-articular or peri-synovial blood flow signal, entheseal thickening, and entheseal erosion. Based on these findings, the presence of synovitis, tenosynovitis, and enthesitis was assessed. Synovitis was defined by synovial thickening with decreased or heterogeneous echogenicity, accompanied by increased blood flow signal within or around the synovium on power Doppler and, in some cases, joint effusion. Tenosynovitis was defined by thickening of the tendon sheath with heterogeneous echogenicity, fluid within the tendon sheath, and increased peritendinous blood flow signal. Enthesitis was defined by entheseal thickening, decreased echogenicity, increased blood flow signal, and/or structural changes such as erosion, cortical irregularity, or osteophyte formation (10).

## Statistical analysis

All statistical analyses were performed using R (version 4.5.1). Normality of continuous variables was assessed with the Shapiro-Wilk test. Normally distributed continuous variables are expressed as mean ± standard deviation (SD) and were compared using the independent-samples t-test (or Welch’s t-test when variances were unequal); non-normally distributed continuous variables are expressed as median (interquartile range) and were compared using the Mann-Whitney U test. Categorical variables are expressed as n (%) and were compared using the chi-square test with Yates’ continuity correction, or Fisher’s exact test when any expected cell count was < 5. Missing data for baseline continuous and categorical variables were addressed using multiple imputation by chained equations (MCE/FCS) in SPSS, generating m = 5 imputed datasets. Multivariable logistic regression models were fitted on each imputed dataset, and final parameter estimates (odds ratios, 95% confidence intervals, and *p* values) were combined using Rubin’s rules. A two-sided *p* value < 0.05 was considered statistically significant.

Independent associated factors for the presence of PsA relative to PsO were identified using multivariable binary logistic regression. Candidate predictors were first screened by univariable logistic regression, and variables with *p* < 0.10 were retained for multivariable modeling. Skewed inflammatory and hepatic markers were log-transformed before entry, and multicollinearity among predictors was assessed using variance inflation factors. Age and sex were force-entered as covariates in an initial full model; a parsimonious final model was then derived by backward elimination, in which age and sex were themselves eligible for removal. Neither age nor sex was retained in either final model. The final models therefore comprised only the variables reported in **Table 1**, and each odds ratio (ORs) is adjusted for the other variables retained in the same model rather than for age and sex. Continuous predictors were standardized to yield ORs per 1-SD increase and re-expressed in clinically interpretable units. CRP and ESR were markedly right-skewed and were therefore log2-transformed (log2[x+1]) prior to model entry, so that the resulting odds ratios directly represent the effect of each doubling in CRP or ESR. Model fit was assessed with the Hosmer-Lemeshow test, discrimination with the area under the receiver-operating-characteristic curve (AUC), and explained variation with the Nagelkerke pseudo-R².

**Table 1:** Multivariable logistic regression of factors independently associated with clinical and subclinical PsA, each compared with PsO.

Variable	Adjusted OR	95% CI	*p* value
Clinical PsA (n = 75) vs. PsO (n = 95)			
Hemoglobin (per 1 g/L)	0.75	0.60–0.90	0.005
Platelet count (per 50x10^9/L)	1.39	1.10–1.78	0.007
CRP (per doubling)	1.78	1.44–2.27	< 0.001
Subclinical PsA (n = 22) vs. PsO (n = 95)			
Hemoglobin (per 10 g/L)	0.65	0.47–0.88	0.008
CRP (per doubling)	1.65	1.21–2.33	0.002

Two separate multivariable logistic regression models were fitted. Age and sex were force-entered into the initial models but were not retained after backward elimination; each odds ratio is therefore adjusted for the other variable(s) shown within the same model. Due to the limited sample size of the subclinical PsA subgroup, platelet count was not assessed in that model. Model fit: clinical PsA model - Hosmer-Lemeshow chi2 = 7.89, *p* = 0.444, AUC = 0.859, Nagelkerke R2 = 0.480; subclinical PsA model - Hosmer-Lemeshow chi2 = 9.00, P = 0.343, AUC = 0.878, Nagelkerke R2 = 0.454. Lymphocyte percentage was excluded from the final model due to collinearity with neutrophil percentage (Spearman’s ρ ≈ −0.87); VIFs for all retained covariates were <2. OR, odds ratio; CI, confidence interval; CRP, C-reactive protein; PsA, psoriatic arthritis; PsO, psoriasis.

## Results

### Demographic and clinical characteristics of patients

From January 2018 to October 2025, 192 patients were admitted to the Shandong Provincial Hospital Affiliated to Shandong First Medical University. The specific process is shown in **Figure 1**. Among them, 75 (39.0%) were classified as clinical PsA patients, 22 (11.5%) as subclinical PsA patients, and 95 (49.5%) as PsO patients. The demographic and clinical characteristics of the PsA (clinical and subclinical PsA) groups and PsO group are summarized in **Table 2**. Compared with the PsO group, patients with PsA trended older, although this difference did not reach statistical significance (median age, 49.0 vs. 45.0 years; *p* = 0.065). Comorbid hypertension was more prevalent in the PsA group (23.7% vs. 11.6%; *p* = 0.044). Notably, the PsA group had a lower percentage of male patients (57.7% vs. 74.7%; *p* = 0.019). No significant differences (*p* > 0.05) were observed between the two groups regarding family history of psoriasis, lifestyle habits (smoking, alcohol consumption), or prior treatment history (conventional, biologic, or small-molecule therapy).

**Figure 1:**
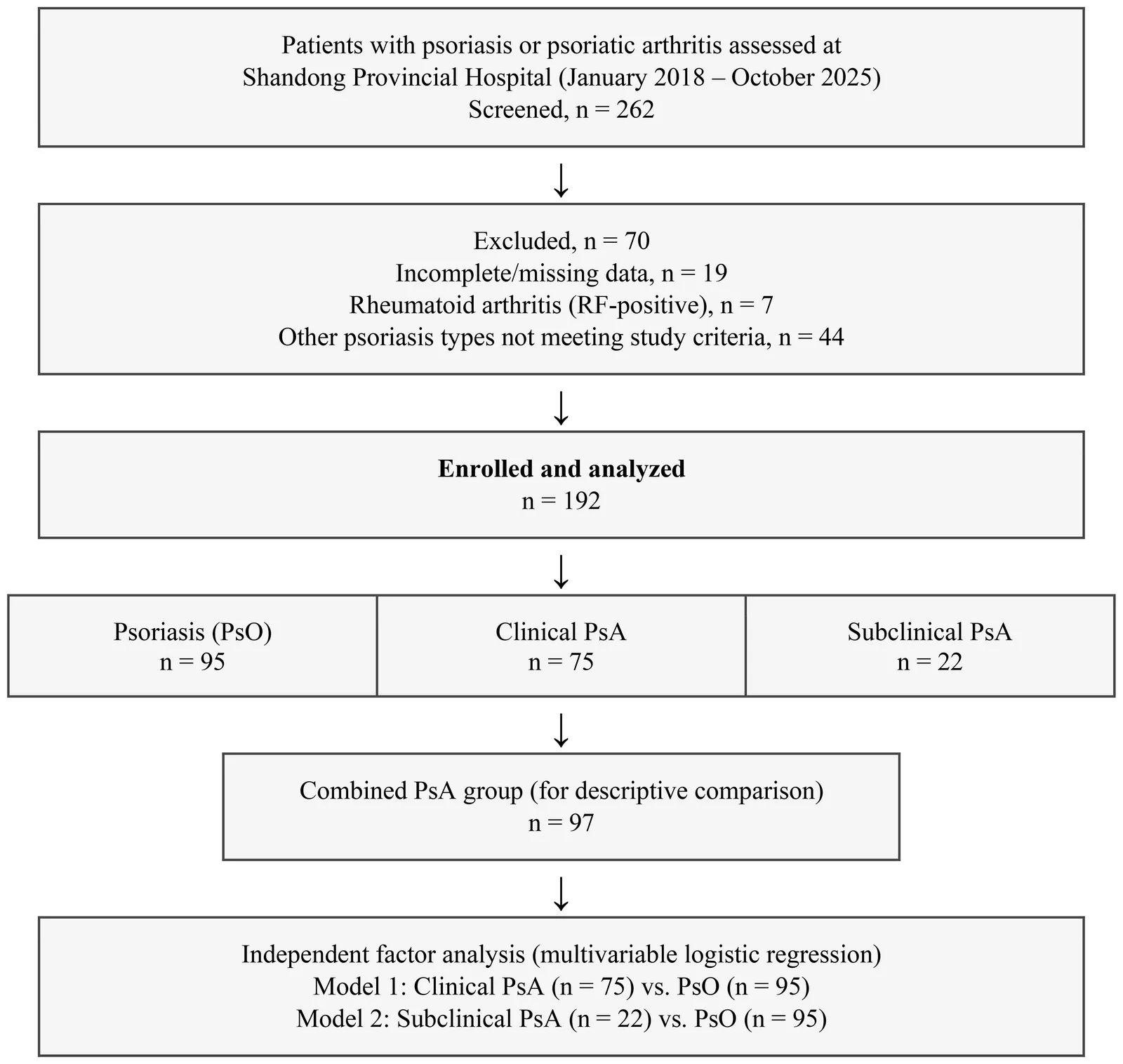
Patient flow diagram.

**Table 2:** Demographic and clinical characteristics of patients with PsA (including clinical and subclinical PsA) and PsO.

Characteristic	PsA (n = 97)	PsO (n = 95)	*p* value
Age, years	49.0 (41.0–55.0)	45.0 (28.5–57.0)	0.065
Age ≥ 40 years, n (%)	74 (76.3)	51 (53.7)	0.002
Duration of psoriasis, years	10.0 (1.0–20.0)	8.0 (2.0–18.5)	0.943
Male sex, n (%)	56 (57.7)	71 (74.7)	0.019
Family history of psoriasis, n (%)	25 (25.8)	20 (21.1)	0.547
Current smoking, n (%)	24 (24.7)	35 (36.8)	0.097
Alcohol consumption, n (%)	30 (30.9)	24 (25.3)	0.476
SBP, mmHg	127.0 (119.0–141.0)	133.0 (122.0–144.0)	0.241
DBP, mmHg	81.85 ± 12.70	81.93 ± 12.86	0.965†
Hypertension, n (%)	23 (23.7)	11 (11.6)	0.044
Cardiovascular disease, n (%)	11 (11.3)	7 (7.4)	0.486
Diabetes mellitus, n (%)	12 (12.4)	7 (7.4)	0.358
Conventional therapy, n (%)	84 (86.6)	90 (94.7)	0.092
Biologic therapy, n (%)	9 (9.3)	5 (5.3)	0.428
Small-molecule drug therapy, n (%)	4 (4.1)	0 (0.0)	0.121*

Data are presented as median (interquartile range) or n (%), as appropriate. Continuous variables were compared using the Mann–Whitney U test; categorical variables were compared using the χ² test with Yates’ continuity correction. *Fisher’s exact test was used when the expected cell count in the 2×2 table was < 5. †Data are presented as mean ± SD and were compared using Student’s t-test (Welch’s t-test used when variances were unequal by Levene’s test). PsA, psoriatic arthritis; PsO, psoriasis vulgaris.

### Laboratory tests of both groups

Compared with the PsO group, patients with PsA had significantly lower HB levels (123.0 vs. 143.0 g/L, *p* < 0.001) and a lower lymphocyte percentage (26.0% vs. 29.0%, *p* = 0.022), but higher PLT counts (301.0 vs. 239.0 ×10^9^/L, *p* < 0.001) and a higher neutrophil percentage (64.0% vs. 59.7%, *p* = 0.004). Patients in the PsA group also exhibited markedly elevated inflammatory markers compared with the PsO group, including CRP (21.4 vs. 2.2 mg/L, *p* < 0.001) and ESR (40.0 vs. 12.5 mm/h, *p* < 0.001). Conversely, albumin levels were significantly reduced in the PsA group (38.9 vs. 40.8 g/L, *p* < 0.001). The laboratory characteristics of the PsA and PsO groups are summarized in **Table 3**.

**Table 3:** Laboratory characteristics of patients with PsA (including clinical and subclinical PsA) and PsO.

Parameter	PsA (n = 97)	PsO (n = 95)	*p* value
WBC, ×10^9^/L	6.63 (5.14–8.99)	6.64 (5.46–7.56)	0.386
Hb, g/L	123.0 (108.0–139.0)	143.0 (133.0–154.0)	< 0.001
Plt, ×10^9^/L	301.0 (245.0–372.1)	239.0 (194.5–290.0)	< 0.001
Lymphocyte, %	26.02 ± 9.28	29.04 ± 8.90	0.022†
Neutrophil, %	64.01 ± 10.97	59.68 ± 9.67	0.004†
Monocyte, %	7.30 (5.80–9.30)	7.20 (6.15–9.45)	0.531
CRP, mg/L	21.41 (5.10–51.21)	2.22 (0.77–5.04)	< 0.001
Albumin, g/L	38.90 (34.50–41.20)	40.80 (38.60–44.25)	< 0.001
ALT, U/L	15.00 (11.00–25.00)	21.00 (15.00–29.50)	0.001
AST, U/L	18.00 (15.00–25.00)	21.00 (19.00–25.50)	0.001
GGT, U/L	26.00 (18.00–39.00)	20.00 (15.00–30.00)	0.015
Fasting glucose, mmol/L	5.24 (4.67–6.00)	4.93 (4.50–5.37)	0.030
Uric acid, µmol/L	286.0 (239.0–361.0)	330.0 (264.0–411.5)	0.008
Creatinine, µmol/L	59.10 (51.80–67.10)	65.90 (59.00–72.55)	0.001
ESR, mm/h	40.00 (20.00–69.00)	12.53 (6.95–23.10)	< 0.001

Data are presented as median (interquartile range) unless otherwise indicated. ^†^Data are presented as mean ± SD and were compared using Student’s t-test (Welch’s t-test used when variances were unequal by Levene’s test); all other variables were compared using the Mann–Whitney U test after Shapiro–Wilk testing indicated non-normal distribution. PsA, psoriatic arthritis; PsO, psoriasis vulgaris; WBC, white blood cell count; Hb, hemoglobin; Plt, platelet count; CRP, C-reactive protein; ALT, alanine aminotransferase; AST, aspartate aminotransferase; GGT, gamma-glutamyl transferase; ESR, erythrocyte sedimentation rate; SBP, systolic blood pressure; DBP, diastolic blood pressure.

Given that demographic and clinical profiles may vary across sex and age, we additionally performed exploratory subgroup analyses stratified by sex (male, female) and age (>40 years); these were hypothesis-generating and were not corrected for multiple comparisons. Overall, the direction of association for the core inflammatory and hematologic markers (CRP, ESR, HB) was broadly consistent with the primary analysis across all three subgroups, supporting the robustness of these findings. Some subgroup-specific differences were also observed: in the female subgroup, hypertension (24.4% vs. 4.2%, *p* = 0.044) and diabetes (19.5% vs. 0%, *p* = 0.022) were more prevalent among patients with PsA; in the male subgroup, a history of alcohol consumption was more common in the PsA group (53.6% vs. 32.4%, *p* = 0.026). Given the limited sample size of these subgroups and the absence of correction for multiple testing, these findings should be interpreted as exploratory and require confirmation in larger, adequately powered studies. Full subgroup results are presented in **Supplementary Tables S1**–**6**.

### Image analysis of both groups

Based on the predefined criteria for PsA, we conducted a comparative analysis of joint involvement and MSUS findings between patients with clinical PsA and subclinical PsA. All enrolled patients underwent comprehensive joint examinations to assess swelling and tenderness in major peripheral joints, with any positive findings recorded as involved joints. The prevalence of individual joint involvement was generally numerically higher in the clinical PsA group, including shoulder involvement (37.3% vs. 13.6%), although these differences did not reach statistical significance (*p* = 0.066 and *p* = 0.064, respectively), likely reflecting the limited sample size of the subclinical PsA group (n = 22). In contrast, MSUS findings showed clear and statistically significant differences: tenosynovitis (17.3% vs. 0%, *p* = 0.036), synovitis (50.7% vs. 0%, *p* < 0.001), synovial thickening (32.0% vs. 4.5%, *p* = 0.021), and joint effusion (30.7% vs. 0%, *p* = 0.007) were all significantly more frequent in patients with clinical PsA (**Table 4**). These findings suggest that ultrasound-detected synovial and tendon pathology may distinguish clinical from subclinical PsA more sensitively than clinical joint examination alone, though this observation warrants confirmation in larger cohorts.

**Table 4:** Joint involvement and musculoskeletal ultrasound findings in patients with clinical and subclinical PsA.

Characteristic	Subclinical PsA (n = 22)	Clinical PsA (n = 75)	*p* value
Proximal interphalangeal joints, n (%)	9 (40.9)	30 (40.0)	1.000
Distal interphalangeal joints, n (%)	12 (54.5)	27 (36.0)	0.189
Metacarpophalangeal joints, n (%)	3 (13.6)	12 (16.0)	1.000*
Wrist joints, n (%)	3 (13.6)	21 (28.0)	0.275
Elbow joints, n (%)	4 (18.2)	13 (17.3)	1.000*
Shoulder joints, n (%)	3 (13.6)	28 (37.3)	0.066
Spine, n (%)	1 (4.5)	18 (24.0)	0.064*
Hip joints, n (%)	4 (18.2)	14 (18.7)	1.000*
Knee joints, n (%)	12 (54.5)	37 (49.3)	0.851
Ankle joints, n (%)	6 (27.3)	24 (32.0)	0.873
Toe joints, n (%)	4 (18.2)	7 (9.3)	0.264*
Proximal toe interphalangeal joints, n (%)	2 (9.1)	9 (12.0)	1.000*
Distal toe interphalangeal joints, n (%)	2 (9.1)	9 (12.0)	1.000*
Osteoarthritis, n (%)	0 (0.0)	10 (13.3)	0.110*
Tendinitis, n (%)	0 (0.0)	3 (4.0)	1.000*
Tenosynovitis, n (%)	0 (0.0)	13 (17.3)	0.036*
Synovitis, n (%)	0 (0.0)	38 (50.7)	< 0.001
Synovial thickening, n (%)	1 (4.5)	24 (32.0)	0.021
Joint effusion, n (%)	0 (0.0)	23 (30.7)	0.007

Data are presented as n (%). Group comparisons were performed using the χ² test with Yates’ continuity correction; ^*^Fisher’s exact test was used when the expected cell count in the 2×2 table was < 5. PsA, psoriatic arthritis. Of the 22 patients classified as subclinical PsA, 21 met the symptom-based criterion only and 1 met both criteria.

### Binary logistic regression

Candidate variables were screened by univariable logistic regression, then entered into multivariable models in which age and sex were force-entered before backward elimination. Because clinical and subclinical PsA differ meaningfully in presentation, we modeled them separately rather than pooling both subtypes into one case group. In the clinical PsA model, lower HB (adjusted OR 0.75 per 10 g/L, 95% CI 0.60–0.90, *p* = 0.005), higher platelet count (adjusted OR 1.39 per 50×10^9^/L, 95% CI 1.10–1.78, *p* = 0.007), and higher CRP (adjusted OR 1.78 per doubling, 95% CI 1.44–2.27, *p* < 0.001) were each independently associated with clinical PsA (**Figure 2**), every estimate being adjusted for the other two variables retained in the model; age and sex did not reach significance, were removed during backward elimination, and were therefore not retained in the final model. HB and CRP showed independent associations of similar or greater magnitude in the subclinical PsA model (n = 22; adjusted OR 0.65, *p* = 0.008; adjusted OR 1.65, *p* = 0.002), while platelet count could not be reliably assessed given the small subgroup size. Both models fit the data well (clinical PsA: Hosmer-Lemeshow *p* = 0.444, AUC = 0.859, Nagelkerke R² = 0.480; subclinical PsA: Hosmer-Lemeshow *p* = 0.343, AUC = 0.878, Nagelkerke R² = 0.454) (**Figure 3**). Complete results are provided in **Table 1**.

**Figure 2:**
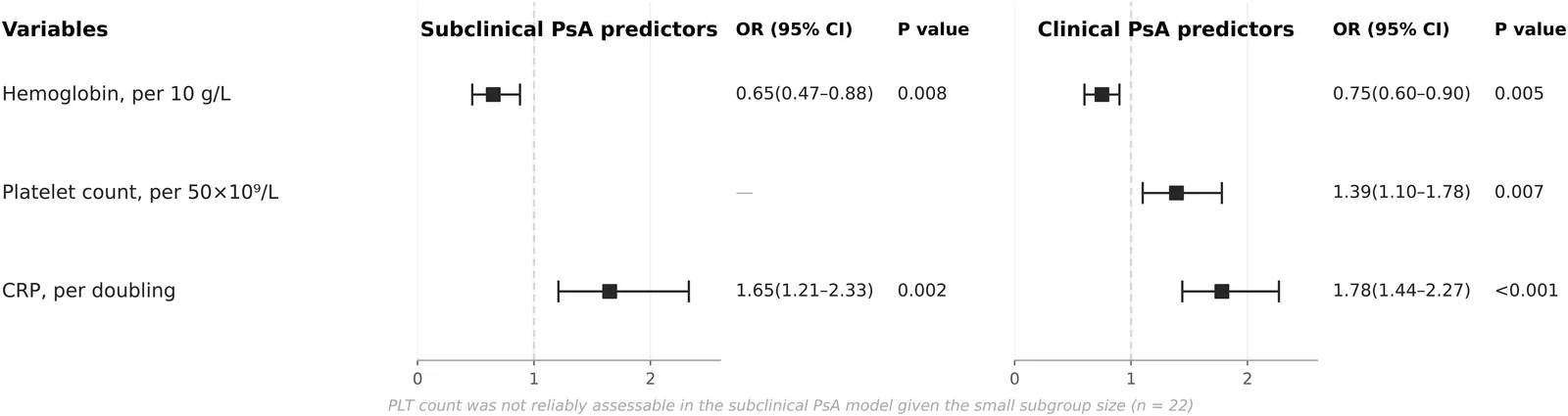
Forest plot of factors independently associated with clinical and subclinical PsA (each vs. PsO). Adjusted odds ratios from separate multivariable logistic regression models, each adjusted for age and sex.

**Figure 3:**
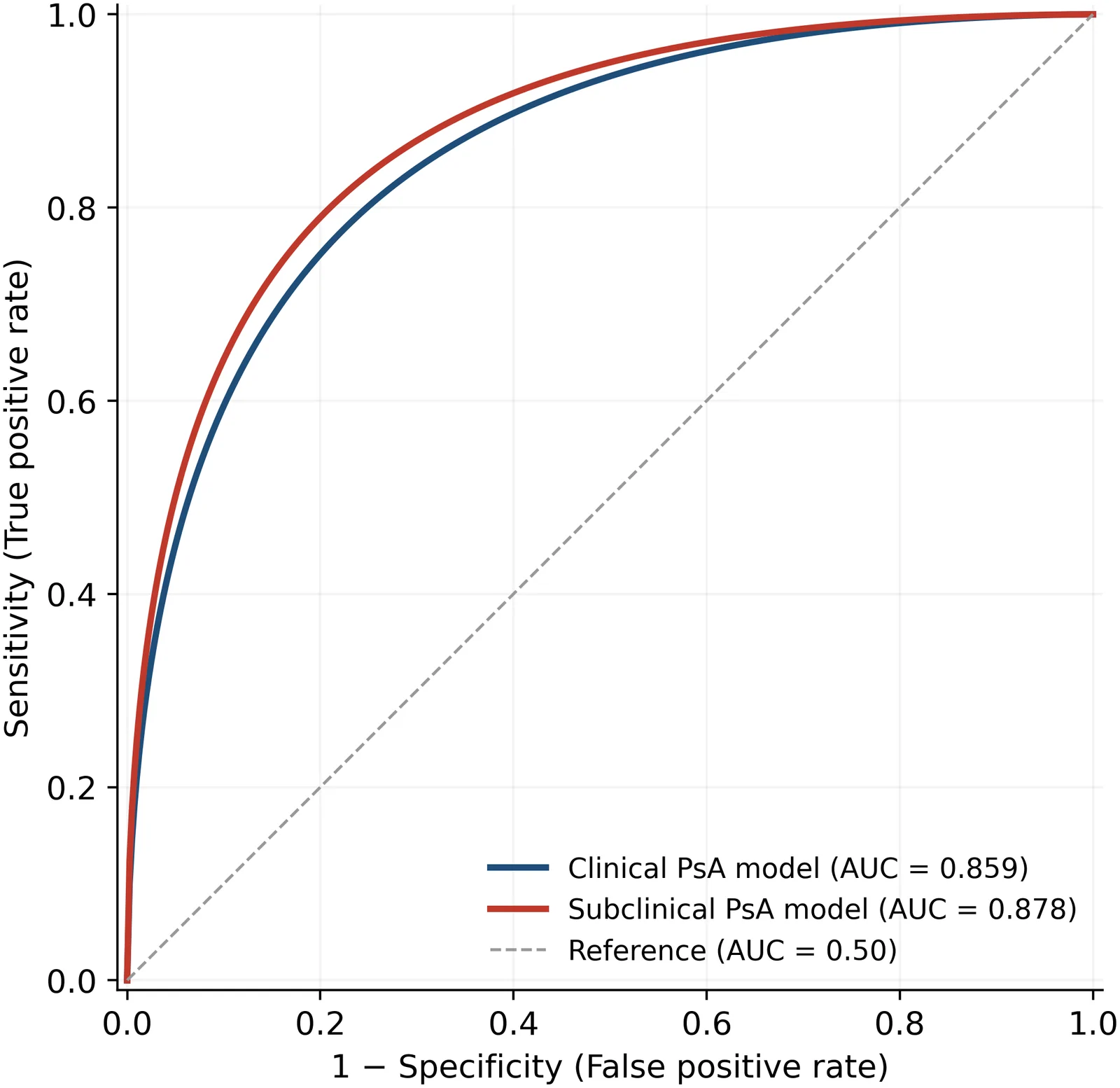
Receiver operating characteristics curves for the clinical and subclinical PsA multivariable models.

## Discussion

In this hospital-based study comparing patients with PsO, subclinical PsA, and clinical PsA, in the clinical PsA model, lower HB, higher platelet count, and higher CRP were each independently associated with the presence of PsA, each estimate being adjusted for the other variables retained in the final model; age and sex were not retained. On MSUS, features of synovial-entheseal inflammation (tenosynovitis, synovitis, synovial hypertrophy, and joint effusion) discriminated clinical from subclinical PsA more effectively than physical examination alone.

The elevated CRP and ESR levels observed in the PsA group in this study are consistent with a state of heightened systemic inflammation, which is thought to arise primarily from dysregulated activation of the IL-23/IL-17 immune axis that is common throughout the psoriatic disease process (11). Th17 cells and innate lymphoid cells residing in the synovial-entheseal complex release inflammatory mediators such as IL-17A, IL-22, and TNF-α, which act on synovial fibroblasts, stromal cells, and bone-related cells to sustain local inflammation. IL-17A and TNF-α also induce RANKL expression and promote osteoclast differentiation via the RANK/NF-κB/NFATc1 signaling pathway, thereby contributing to bone erosion. This inflammatory network simultaneously upregulates IL-6 expression, which in turn drives hepatic CRP synthesis (12), whereas ESR primarily reflects changes in fibrinogen and other acute-phase proteins. Accordingly, the elevated CRP and ESR levels observed in our PsA patients are best interpreted as markers of an increased overall systemic inflammatory burden accompanying psoriatic inflammation with joint involvement, rather than as indicators specific to localized synovitis. Damage-associated molecular patterns released during enthesitis and bone remodeling, such as calprotectin, may further amplify this inflammatory state through TLR4/RAGE-dependent innate immune activation. HB level showed a clinically meaningful reduction in the PsA group, with its lower quartile falling below the standard anemia threshold, consistent with features of anemia of chronic inflammation (13). Under chronic inflammatory conditions, cytokine-driven upregulation of hepcidin limits intestinal iron absorption and promotes iron sequestration within the reticuloendothelial system, resulting in iron-restricted erythropoiesis. Notably, HB remained independently associated with PsA after multivariable adjustment, suggesting that it could carry independent discriminative value. By contrast, lymphocyte percentage and albumin lost statistical significance once CRP and HB were included in the model, suggesting that their associations in univariate analysis may largely reflect a shared underlying inflammatory burden rather than factors specifically and independently linked to joint involvement.

In chronic immune-mediated inflammatory diseases, activated PLT release a variety of inflammatory mediators and form PLT–leukocyte aggregates with neutrophils and monocytes, promoting the recruitment of inflammatory cells and endothelial activation (14), and thereby contributing to the ongoing amplification of the inflammatory response. Under inflammatory conditions, IL-6 promotes hepatic synthesis of thrombopoietin, enhancing megakaryocyte proliferation and PLT production and leading to reactive thrombocytosis. Accordingly, an elevated circulating PLT with elevated CRP and ESR, may jointly reflect an increased systemic inflammatory burden associated with PsO. In this study, an elevated PLT was independently associated with higher odds of PsA (adjusted OR 1.39 per 50 × 10^9^/L increase in PLT). Prior studies have shown that PLT correlates with the systemic inflammatory state in patients with PsO and PsA and may serve as a simple hematologic marker reflecting disease-related inflammatory burden. However, PLT did not show a consistent correlation with local inflammatory imaging markers such as MSUS detected synovial blood-flow signals, suggesting that PLT more likely reflects overall systemic inflammatory burden rather than specifically representing local joint inflammatory activity. Therefore, in patients with PsO, an elevated PLT may indicate a persistently heightened immune-inflammatory background and could hold value as an ancillary marker associated with concurrent joint involvement; because our data were collected at a single time point, this association cannot be interpreted as indicating risk of subsequently developing PsA.

The proportion of shoulder and spinal involvement was higher among patients with clinical PsA than subclinical PsA, although this difference did not reach statistical significance, likely reflecting the small sample size of the subclinical PsA group (n = 22). More extensive joint involvement is typically observed in patients with PsO of longer disease duration and greater disease burden, suggesting that the two groups may occupy different positions along the spectrum of PsO-related joint disease (15). However, we cannot determine whether patients with subclinical disease will progress to clinically overt PsA over time; this remains speculative and awaits confirmation in longitudinal studies. Synovitis was substantially more prevalent in the clinical PsA group than in the subclinical group, a difference that was highly significant. Tenosynovitis, synovial hypertrophy, and joint effusion followed the same pattern, each occurring markedly more often among patients with clinical PsA. Osteoarthritis was also numerically more frequent in the clinical PsA group, though these differences did not reach statistical significance, likely because the small sample size of the subclinical group limited statistical power. This is consistent with previous imaging studies showing that subclinical synovial-entheseal inflammation is not uncommon among patients with PsO who have not yet developed overt arthritis (16), and that MSUS can capture this stage of psoriatic arthralgia; MSUS-based phenotyping has also been used to refine the classification of psoriatic musculoskeletal disease (17). In our cohort, 21 of the 22 patients so labeled were classified on musculoskeletal symptoms alone and only 1 showed an ultrasound abnormality. This comparison should accordingly be regarded as a descriptive finding rather than evidence supporting the validity of the subclinical PsA classification.

Our findings are directionally consistent with two of the most closely related recent studies, although the differences in effect size merit further discussion. In the Toronto prospective PsO cohort, higher high-sensitivity CRP (hs-CRP) levels were found to predict the subsequent development of PsA, confirming a temporal relationship between inflammation and disease onset (18), a meta-analysis of candidate biomarkers likewise identified CRP and ESR as the most reproducible serologic markers associated with PsA (19). The adjusted odds ratios in our clinical PsA model were considerably higher than the hazard ratios reported in the prospective cohort above, and this discrepancy more likely reflects differences in study design than differences in the underlying populations. Hospitalized patients with confirmed, active PsA inherently have higher levels of acute-phase reactants, so this study would be expected to amplify the observed effect. Our results are also corroborated to some extent by other cohorts within China. Huang et al. constructed a composite score based on serum inflammatory markers to help identify PsA among patients with PsO (20), and Wang et al. developed an ultrasound-based risk model for early detection of PsA (21). Our findings are directionally consistent with both of these studies, while also suggesting that serologic and ultrasound signals are not interchangeable but complementary: MSUS abnormalities effectively distinguished clinical from subclinical PsA even when clinical joint counts could not. Regarding sex, several systematic reviews and cohort studies have reported that female sex and age over 40 years are associated with the development of PsA (22), and in our unadjusted comparison, the proportion of women was indeed higher in the PsA group. However, sex was no longer independently associated with PsA after multivariable adjustment, indicating that our data do not support an independent sex effect. The published evidence on this point is itself not entirely consistent: sex differences in PsA phenotype, symptom reporting, and treatment response have been reported, yet the PRESTO risk-assessment tool identifies male sex as one of the factors associated with short-term risk of developing PsA (23). Sex-related effects in psoriasis may be influenced by multiple factors, including referral pathways, disease duration, and outcome definitions, and a single-center hospitalized cohort cannot provide a definitive conclusion on this point. With regard to age, in the subgroup older than 40 years we observed that the mean age of the psoriasis control group was higher than that of the PsA group, consistent with the well-recognized bimodal age distribution of psoriasis onset (24). Late-onset psoriasis typically peaks in the late 50 to 60, tends to follow a relatively stable cutaneous course, and less often involves the musculoskeletal system, whereas psoriatic arthritis itself typically develops between 40 and 50 years of age. This suggests that the higher mean age observed in the older psoriasis subgroup may reflect a relatively greater proportion of patients with late-onset, joint-uninvolved psoriasis. It should be noted that this interpretation is drawn from an exploratory, unadjusted subgroup analysis and remains speculative, requiring further validation.

Even a six-month delay in the diagnosis of PsA is associated with greater peripheral joint erosion and worse long-term physical function (25), and nonspecific musculoskeletal symptoms often precede formal diagnosis (26). Our data suggest a low-cost, stratified screening strategy applicable to both inpatient and outpatient dermatology settings: among patients with PsO who present with arthralgia, morning stiffness, or functional limitation, clinicians should raise their index of suspicion—and should prioritize musculoskeletal MSUS rather than simply attributing symptoms to degenerative or mechanical causes—when CRP, ESR, or PLT count is also elevated. It should be emphasized that these markers are not specific to psoriatic joint disease; infection, malignancy, obesity, and other comorbidities can likewise elevate acute-phase reactants. These indicators should therefore be used as adjunctive evidence—in the context of an already-suspicious clinical presentation — to help decide which patients with PsO warrant musculoskeletal assessment for coexisting joint involvement, rather than as an independent screening tool or as an indicator of future progression to PsA.

This study has several limitations that warrant specific discussion. First, this was a single-time-point retrospective study rather than a longitudinal one. Because exposure and outcome data were collected at the same time point, we cannot establish a temporal relationship between the two, and we therefore cannot exclude the possibility that the observed abnormalities in inflammatory markers were in fact a consequence of pre-existing joint inflammation rather than a precursor to it. Second, all participants were hospitalized patients from a single center, and the subclinical PsA group included only 22 patients, a relatively limited sample size. Compared with community or outpatient populations, hospitalized patients with PsO tend to have more extensive skin involvement, more comorbidities, and inherently higher baseline acute-phase reactant levels, which may have amplified the between-group differences; moreover, classification of the subclinical group relied on symptom reporting and MSUS findings, introducing potential detection and verification bias. The small sample size not only limited statistical power and widened confidence intervals but also constrained the number of covariates that could be included simultaneously in the models and increased the risk of model instability in the subclinical analyses. Third, prior anti-inflammatory or immunomodulatory treatment may have unevenly reduced measured acute-phase reactant levels among patients. Residual confounding from this source is therefore likely, and the associations reported here should be understood as not having been adjusted for these factors, rather than as findings independent of them. Fourth, the methodology of MSUS assessment may have affected the interpretation of our findings. Ultrasound was not performed systematically in asymptomatic patients, and therefore the PsO comparison group was not routinely screened for subclinical synovio-entheseal inflammation, potentially resulting in misclassification of some patients with subclinical PsA. In addition, our operational definition of subclinical PsA, based on the presence of either musculoskeletal symptoms or MSUS abnormalities rather than requiring both, was broader than that used in some previous cohorts and may have included a more heterogeneous population. In particular, only 1 of the 22 patients in this group had objective ultrasound evidence of synovio-entheseal involvement. However, because the study focused primarily on the major peripheral joints and did not assess smaller joints such as the temporomandibular joint, the extent of synovio-entheseal involvement may have been underestimated. All subgroup analyses were exploratory in nature, and those not corrected for multiple testing should be regarded as hypothesis-generating only, pending confirmation in future studies. Despite these limitations, to our knowledge this study is the first to jointly evaluate laboratory markers and MSUS features across clinical and subclinical PsA within the same cohort, providing a hypothesis-generating foundation for future prospective validation and offering a practical, preliminary basis for identifying patients with PsO in whom coexisting joint involvement is more likely and who may therefore warrant musculoskeletal assessment in dermatologic practice.

## Conclusions

Lower HB and elevated CRP were independently and consistently associated with PsA across both clinical and subclinical subgroups, with PLT count showing an additional independent association in clinical PsA. These readily available hematologic and inflammatory markers, particularly when combined with musculoskeletal ultrasound, may help dermatologists identify psoriasis patients who warrant earlier rheumatological referral. For this study, prospective longitudinal research is needed to confirm whether these markers can predict incident PsA.

## Data Availability

The raw data supporting the conclusions of this article will be made available by the authors, without undue reservation.
