# Supplementary material for: Clinical, hematologic, and inflammatory factors associated with psoriatic arthritis in a Chinese hospital-based study
Source: Front Immunol. 2026 Sep 15;17:1928389. doi: 10.3389/fimmu.2026.1928389 (PMC13619816; doi:10.3389/fimmu.2026.1928389)
Supplement: Supplementary file 1 [file Table1.docx]

Supplementary Table 1. Demographic and Clinical Characteristics of Male Patients with PsA (Including Clinical and Subclinical PsA) and PsO.

| **Characteristic** | **PsA (n = 56)** | **PsO (n = 71)** | ***p* value** |
| --- | --- | --- | --- |
| Age, years | 48.50 (39.75–54.25) | 45.00 (31.00–56.50) | 0.277^c^ |
| Age ≥ 40 years, n (%) | 42 (75.0) | 38 (53.5) | 0.021^d^ |
| Duration of psoriasis, years | 10.00 (2.00–20.00) | 10.00 (3.00–19.50) | 0.755^c^ |
| Family history of psoriasis, n (%) | 11 (19.6) | 15 (21.1) | 1.000^d^ |
| Current smoking, n (%) | 24 (42.9) | 35 (49.3) | 0.587^d^ |
| Alcohol use, n (%) | 30 (53.6) | 23 (32.4) | 0.026^d^ |
| SBP, mmHg | 130.00 (121.50–138.25) | 135.00 (124.00–144.00) | 0.117^c^ |
| DBP, mmHg | 81.25 ± 11.99 | 83.27 ± 12.67 | 0.364^a^ |
| Hypertension, n (%) | 13 (23.2) | 10 (14.1) | 0.274^d^ |
| Cardiovascular disease, n (%) | 6 (10.7) | 7 (9.9) | 1.000^d^ |
| Diabetes mellitus, n (%) | 4 (7.1) | 7 (9.9) | 0.754^e^ |
| Conventional therapy, n (%) | 47 (83.9) | 67 (94.4) | 0.103^d^ |
| Biologic therapy, n (%) | 6 (10.7) | 4 (5.6) | 0.334^e^ |
| Small-molecule therapy, n (%) | 3 (5.4) | 0 (0.0) | 0.083^e^ |

Data are presented as median (interquartile range) or n (%). ^a^Student's t-test (independent samples, equal variances); ^c^Mann-Whitney U test; ^d^Pearson's chi-square test (with continuity correction); ^e^Fisher's exact test (used when any expected cell count < 5). PsA, psoriatic arthritis; PsO, psoriasis vulgaris.

Supplementary Table 2. Laboratory Characteristics of Male Patients with PsA (Including Clinical and Subclinical PsA) and PsO.

| **Parameter** | **PsA (n = 56)** | **PsO (n = 71)** | ***p* value** |
| --- | --- | --- | --- |
| WBC, ×10⁹/L | 6.97 (5.49–9.07) | 6.78 (5.76–7.55) | 0.531^c^ |
| Hb, g/L | 127.00 (108.00–142.00) | 147.00 (139.00–157.00) | <0.001^c^ |
| Plt, ×10⁹/L | 306.00 (236.25–372.58) | 232.00 (194.50–284.00) | <0.001^c^ |
| Lymphocyte, % | 24.75 ± 8.10 | 28.29 ± 8.21 | 0.017^a^ |
| Neutrophil, % | 65.04 ± 9.58 | 59.76 ± 9.34 | 0.002^a^ |
| Monocyte, % | 8.25 (6.45–10.12) | 7.70 (6.35–9.65) | 0.791^c^ |
| CRP, mg/L | 19.95 (6.10–51.55) | 2.56 (0.77–5.97) | <0.001^c^ |
| Albumin, g/L | 37.85 (34.03–40.73) | 40.60 (38.60–44.00) | <0.001^c^ |
| ALT, U/L | 16.50 (11.00–25.25) | 24.00 (17.00–31.50) | 0.004^c^ |
| AST, U/L | 17.00 (15.00–21.25) | 23.00 (20.00–26.00) | <0.001^c^ |
| GGT, U/L | 30.50 (22.75–44.75) | 22.00 (17.50–37.00) | 0.006^c^ |
| Glucose, mmol/L | 5.15 (4.67–6.02) | 4.96 (4.52–5.37) | 0.030^c^ |
| Uric acid, µmol/L | 317.69 ± 100.10 | 369.24 ± 105.53 | 0.006^a^ |
| Creatinine, µmol/L | 62.23 (55.09–72.38) | 68.60 (62.40–76.75) | 0.014^c^ |
| ESR, mm/h | 46.00 (22.75–78.50) | 12.53 (6.45–21.94) | <0.001^c^ |

Data are presented as mean ± SD or median (interquartile range), depending on the distribution assessed by the Shapiro-Wilk test. ^a^Student's t-test (independent samples, equal variances); ^c^Mann-Whitney U test. PsA, psoriatic arthritis; PsO, psoriasis vulgaris; SBP, systolic blood pressure; DBP, diastolic blood pressure; WBC, white blood cell count; Hb, hemoglobin; Plt, platelet count; CRP, C-reactive protein; ALT, alanine aminotransferase; AST, aspartate aminotransferase; GGT, gamma-glutamyl transferase; ESR, erythrocyte sedimentation rate.

Supplementary Table 3. Demographic and Clinical Characteristics of Female Patients with PsA (Including Clinical and Subclinical PsA) and PsO.

| **Characteristic** | **PsA (n = 41)** | **PsO (n = 24)** | ***p* value** |
| --- | --- | --- | --- |
| Age, years | 50.0 ± 13.4 | 41.5 ± 22.4 | 0.101^*^ |
| Age ≥ 40 years, n (%) | 32 (78.0) | 13 (54.2) | 0.083 |
| Family history of psoriasis, n (%) | 14 (34.1) | 5 (20.8) | 0.392 |
| Current smoking, n (%) | 0 (0.0) | 0 (0.0) | NA |
| Alcohol consumption, n (%) | 0 (0.0) | 1 (4.2) | 0.369^†^ |
| Hypertension, n (%) | 10 (24.4) | 1 (4.2) | 0.044^†^ |
| Cardiovascular disease, n (%) | 5 (12.2) | 0 (0.0) | 0.149^†^ |
| Diabetes mellitus, n (%) | 8 (19.5) | 0 (0.0) | 0.022^†^ |
| Conventional therapy, n (%) | 37 (90.2) | 23 (95.8) | 0.644^†^ |
| Biologic therapy, n (%) | 3 (7.3) | 1 (4.2) | 1.000^†^ |
| Small-molecule drug therapy, n (%) | 1 (2.4) | 0 (0.0) | 1.000^†^ |

Data are presented as mean ± SD or n (%), as appropriate. Age was compared using Student's t-test with Welch's correction applied due to unequal variances; categorical variables were compared using the χ² test with Yates' continuity correction. ^†^Fisher's exact test was used when the expected cell count in the 2 × 2 table was < 5. ^*^Compared using Student's t-test (Welch's correction applied where variances were unequal). NA, not applicable (no events in either group). PsA, psoriatic arthritis; PsO, psoriasis vulgaris.

Supplementary Table 4. Laboratory Characteristics of Female Patients with PsA (Including Clinical and Subclinical PsA) and PsO.

| **Parameter** | **PsA (n = 41)** | **PsO (n = 24)** | ***p* value** |
| --- | --- | --- | --- |
| WBC, ×10⁹/L | 6.8 ± 2.6 | 5.9 ± 1.8 | 0.128^*^ |
| Hb, g/L | 118.0 (106.0–130.0) | 128.0 (122.0–135.2) | 0.031 |
| Plt, ×10⁹/L | 293.0 (245.0–363.3) | 245.0 (208.0–296.0) | 0.006 |
| Lymphocyte, % | 27.7 ± 10.5 | 31.3 ± 10.6 | 0.200^*^ |
| Neutrophil, % | 62.6 ± 12.6 | 59.4 ± 10.8 | 0.287^*^ |
| Monocyte, % | 6.3 (5.1–8.4) | 6.9 (5.7–7.4) | 0.844 |
| CRP, mg/L | 21.4 (3.1–46.2) | 1.5 (0.8–2.5) | <0.001 |
| Albumin, g/L | 39.2 ± 5.4 | 41.3 ± 3.3 | 0.058^*^ |
| ALT, U/L | 14.0 (11.0–23.8) | 16.5 (13.8–21.2) | 0.189 |
| AST, U/L | 19.0 (15.6–26.0) | 19.0 (17.8–22.2) | 0.935 |
| GGT, U/L | 20.0 (16.0–30.0) | 15.0 (11.0–20.0) | 0.029 |
| Glucose, mmol/L | 5.3 (4.5–5.8) | 4.9 (4.4–5.5) | 0.514 |
| Uric acid, µmol/L | 256.1 (229.0–303.0) | 251.5 (206.5–317.8) | 0.744 |
| Creatinine, µmol/L | 53.5 (48.4–61.5) | 52.5 (45.9–60.7) | 0.881 |
| ESR, mm/h | 39.0 (14.0–57.0) | 13.1 (7.1–28.3) | 0.003 |
| SBP, mmHg | 125.0 (114.0–146.0) | 122.5 (111.8–139.0) | 0.480 |
| DBP, mmHg | 82.7 ± 13.7 | 78.0 ± 12.8 | 0.171^*^ |

Data are presented as median (interquartile range) unless otherwise indicated. ^*^Data are presented as mean ± SD and were compared using Student's t-test (Welch's correction applied where variances were unequal); all other variables were compared using the Mann–Whitney U test after Shapiro–Wilk testing indicated non-normal distribution. PsA, psoriatic arthritis; PsO, psoriasis vulgaris; WBC, white blood cell count; Hb, hemoglobin; Plt, platelet count; CRP, C-reactive protein; ALT, alanine aminotransferase; AST, aspartate aminotransferase; GGT, gamma-glutamyl transferase; ESR, erythrocyte sedimentation rate; SBP, systolic blood pressure; DBP, diastolic blood pressure.

Supplementary Table 5. Demographic and Clinical Characteristics of Patients Aged >40 Years with PsA (Including Clinical and Subclinical PsA) and PsO.

| **Characteristic** | **PsA (n = 74)** | **PsO (n = 51)** | ***p* value** |
| --- | --- | --- | --- |
| Age, years | 52.50 (47.25–58.75) | 56.00 (53.00–62.00) | 0.013 |
| Duration of psoriasis, years | 10.00 (2.00–20.00) | 12.00 (6.00–20.00) | 0.120 |
| Male sex, n (%) | 42 (56.8) | 38 (74.5) | 0.065 |
| Family history of psoriasis, n (%) | 16 (21.6) | 10 (19.6) | 0.961 |
| Current smoking, n (%) | 20 (27.0) | 21 (41.2) | 0.144 |
| Alcohol consumption, n (%) | 25 (33.8) | 16 (31.4) | 0.930 |
| Hypertension, n (%) | 22 (29.7) | 11 (21.6) | 0.417 |
| Cardiovascular disease, n (%) | 11 (14.9) | 6 (11.8) | 0.817 |
| Diabetes mellitus, n (%) | 11 (14.9) | 7 (13.7) | 1.000 |
| Conventional therapy, n (%) | 63 (85.1) | 47 (92.2) | 0.364 |
| Biologic therapy, n (%) | 7 (9.5) | 4 (7.8) | 1.000^*^ |
| Small-molecule drug therapy, n (%) | 4 (5.4) | 0 (0.0) | 0.145^*^ |

Data are presented as median (interquartile range) or n (%). Continuous variables were compared using the Mann-Whitney U test; categorical variables were compared using the χ² test with Yates' continuity correction. ^*^Fisher's exact test was used when the expected cell count in the 2 × 2 table was < 5. PsA, psoriatic arthritis; PsO, psoriasis vulgaris.

Supplementary Table 6. Laboratory Characteristics of Patients Aged >40 Years with PsA (Including Clinical and Subclinical PsA) and PsO.

| **Parameter** | **PsA (n = 74)** | **PsO (n = 51)** | ***p* value** |
| --- | --- | --- | --- |
| WBC, ×10⁹/L | 6.82 (5.22–8.92) | 6.16 (5.19–7.05) | 0.074 |
| Hb, g/L | 121.50 (108.00–139.00) | 139.00 (126.00–148.50) | <0.001 |
| Plt, ×10⁹/L | 303.00 (241.00–365.32) | 229.00 (195.00–269.00) | <0.001 |
| Lymphocyte, % | 25.06 ± 8.92 | 28.27 ± 8.90 | 0.050^†^ |
| Neutrophil, % | 64.77 ± 10.44 | 60.22 ± 9.72 | 0.015^†^ |
| Monocyte, % | 7.24 (5.80–9.15) | 7.30 (6.80–9.65) | 0.106 |
| CRP, mg/L | 22.74 (5.51–52.21) | 2.69 (1.40–6.82) | <0.001 |
| Albumin, g/L | 37.80 (33.97–40.35) | 39.70 (37.55–41.60) | 0.011 |
| ALT, U/L | 15.00 (11.00–24.93) | 20.00 (14.00–26.50) | 0.047 |
| AST, U/L | 18.00 (15.00–24.75) | 21.00 (18.00–25.50) | 0.024 |
| GGT, U/L | 26.00 (18.25–39.75) | 20.00 (16.00–29.50) | 0.036 |
| Glucose, mmol/L | 5.38 (4.78–6.05) | 4.90 (4.50–5.39) | 0.014 |
| Uric acid, µmol/L | 289.32 ± 91.06 | 338.49 ± 109.97 | 0.007^†^ |
| Creatinine, µmol/L | 58.68 (50.70–66.18) | 66.00 (59.50–72.55) | 0.001 |
| ESR, mm/h | 45.50 (23.00–72.25) | 17.00 (9.61–23.33) | <0.001 |
| SBP, mmHg | 129.50 (119.00–146.00) | 138.00 (128.00–152.00) | 0.070 |
| DBP, mmHg | 83.16 ± 12.42 | 83.71 ± 15.18 | 0.827^†^ |

Data are presented as median (interquartile range) unless otherwise indicated. ^†^Data are presented as mean ± SD and were compared using the independent-samples t-test; all other continuous variables were compared using the Mann-Whitney U test after Shapiro-Wilk testing indicated non-normal distribution. WBC, white blood cell count; Hb, hemoglobin; Plt, platelet count; CRP, C-reactive protein; ALT, alanine aminotransferase; AST, aspartate aminotransferase; GGT, gamma-glutamyl transferase; ESR, erythrocyte sedimentation rate; SBP, systolic blood pressure; DBP, diastolic blood pressure.
